# Paediatric Major Trauma Psychology Service Evaluation: An Early Review of an Integrated Model of Care

**DOI:** 10.3390/children12020241

**Published:** 2025-02-18

**Authors:** Rebecca Bundy, Jade Taktak, Zoe Berger, Ewa Nowotny, Idit Albert

**Affiliations:** Paediatric Psychological Therapies Service, St George’s Hospital NHS Trust, London SW17 0QT, UKzoe.berger@stgeorges.nhs.uk (Z.B.);

**Keywords:** major trauma, PTSD, paediatric mental health

## Abstract

Background: Major trauma is a leading cause of death and disability in children and young people (CYP) in the United Kingdom (UK). Since 2012, major trauma centres (MTCs) have been created with specialist expertise to treat patients suffering with lifechanging injuries. Much research has focused on the physical recovery of patients; however, the psychological and psychosocial impacts of major trauma are significant and often neglected/deprioritised. Less is known about this area in relation to a paediatric population. Methods: This service evaluation reports on the first year of an integrated psychological pathway within a London paediatric MTC. The proactive approach involves inpatient and outpatient psychological support, screening children and families for trauma symptoms and emotional distress, offering psychological intervention where required, and liaison with community mental health teams, social care services and third sector organisations. Descriptive statistics are reported on the patient demographics and mechanism of injury, as well as screening scores at 6 weeks and 3 months following the traumatic incident. Patient feedback is also presented. Results: The results demonstrate a significant increase in the numbers of children and families offered psychological support following the creation of the pathway and an overwhelmingly positive response from service users. Conclusions: Clinical implications are outlined, and areas for further development are discussed.

## 1. Introduction

Major trauma is the leading cause of death and disability in children and young people in the UK [1,2].

Medically, major trauma describes injuries that affect single areas of the body or multiple body systems that are life-threatening and often lifechanging as they may lead to long-term disability [3]. Examples of events causing major trauma might include road traffic accidents, falls, sport injuries, or violent assaults, such as knife crime.

Since 2010, healthcare for major trauma patients in the UK and Ireland has been reorganised, and the creation of major trauma centres (MTCs) occurred in 2012. MTCs centralise specialist expertise and facilities to treat patients suffering with severe and life-threatening injuries with the explicit intention of improving patient outcomes and optimising the delivery of clinical care [4]. With these improvements to care pathways, the survival rate for major trauma is increasing [5], but it is important to note that recovery is often lengthy, and patients are required to navigate changes to their health status and functioning over the longer term.

Much of the research has focused on the physical impact and recovery of patients. However, the psychological and psychosocial impacts of major trauma are far reaching [6,7]. It is reported that around a third of trauma survivors develop depression, and more than a quarter develop post-traumatic stress disorder (PTSD) [8]. There are further psychosocial impacts on social and family life, including the wellbeing of families and carers, as well as on education and work. A recent survey of current practices for major trauma care across the UK and Ireland has found that psychological and psychosocial care following major trauma is often inequitable across the trauma care pathways [6].

There is a good understanding within paediatric settings that parents of children and young people admitted may experience psychological distress during acute hospital stays, and this can impact negatively on children’s outcomes [9,10]. Cross-sectional evidence reports higher rates of PTSD symptoms in children admitted to paediatric intensive care units (PICUs), and symptoms are positively correlated with parents [11]. One study found nearly half of families of children admitted to PICUs experienced significant symptoms of PTSD 12 months following discharge [12]. With this in mind, a systemic approach in which the psychological care of a young person extends to their system of concern, within paediatric settings, is vital.

In terms of the treatment of symptoms, it is well known that psychological intervention for trauma can be very effective [13]. In line with the NICE guidelines, active monitoring is suggested for people with subthreshold symptoms within one month of a traumatic event [14]. An individual trauma intervention is recommended if symptoms persist between 1 and 3 months after the traumatic event, for children under 18 years old. Guidelines recommend trauma-focused cognitive behaviour therapy (TF-CBT) as a first-line treatment, followed by eye movement desensitisation and reprocessing (EMDR) (if symptoms persist for more than 3 months). Both treatment approaches have growing evidence bases showing their effectiveness for treating trauma [13,15].

### Local Context

In 2010, the Greater London area became the first area of the UK to implement a regional trauma system. St. George’s Hospital NHS Trust is one of four hospitals in London that achieved the high standard necessary to become a designated Major Trauma Centre.

Despite significant numbers of patients, access to early psychological support for paediatric patients and their families remained variable and often insufficient.

In 2019, NHS London’s violence reduction programme explored the pathways that look at the psychological support available from mental health services and Major Trauma Centres (MTCs) for victims of violence. This work again illustrated the disparity of care between psychological and physical health pathways for victims of major trauma.

The findings led to the establishment of the London Major Trauma Psychology Network in 2023 and to the development of a Major Trauma Integrated Psychological Model of Care within London’s four major trauma centres (paediatric and adult). Unlike other models of care, the Pan-London Major Trauma Psychology Pathway incorporates mental health support into routine patient care. Major trauma patients are proactively assessed on the ward, regardless of whether they present with mental health needs. This allows for the identification of emotional support needs that might otherwise be missed, particularly for patients who may struggle with emotional expression or who may have communication difficulties. It also enables increased equity of access to our services and resources. 

The hospital secured funding for one clinical psychologist (0.8 WTE) and one assistant psychologist (1.0 WTE) to provide psychological support for children and young people admitted to the Major Trauma Centre. These two posts sat within a larger well-established Paediatric Psychological Therapies Service that offers specialist inpatient and outpatient psychological assessments and interventions to CYP under the care of a number of medical specialties.

This article presents information about the integrated model within paediatrics as well as the data collected over the first 12 months of the Major Trauma Integrated Psychological Model of Care Pilot.

## 2. Materials and Methods

### 2.1. Cohort

This is a retrospective service evaluation of the psychological screening and support provided to children under the age of 18 years, admitted to a paediatric major trauma centre at one of the tertiary centre pilot sites in London, UK, between July 2023 and July 2024. Data reported are for children who were admitted to a ward for an inpatient stay. Children who attended the hospital but were not admitted were not captured in this pilot. Demographic (age, gender, and ethnicity) and mechanism of injury data were obtained from the young people’s medical records and clinical interviews.

### 2.2. Acceptability of the Model

The purpose of this paper is to describe the integrated pathway that was developed, utilisation of the service and to comment on the acceptability of the model to service users.

### 2.3. Ethics

A full ethics review, under the terms of the Government Arrangements of Research Ethics Committees in the UK, was not required, as the data analysis was retrospective, and no additional data were collected beyond that collected for standard medical care. A service evaluation was registered with the NHS Trust Audit Committee (registration number AUDI004088).

### 2.4. Development of an Integrated Model of Care

The integrated model of care offers a proactive preventative approach to children and families, whereby all the children admitted to a ward under the major trauma team are offered psychological assessment, monitoring, and intervention, if needed, as a part of standard care. Several factors were taken into account when considering the development of the service. These included normalising and destigmatising access to psychological professionals, validating the varied emotional responses to physical trauma and pain, consideration of the systemic nature of working within a paediatric setting and the NICE guidelines for treating psychological trauma to include active monitoring and treatment following a traumatic event [14].

Learning was based on an early pilot phase of the project, in 2023, which has aligned with research coming out of the US relating to acute paediatric trauma settings [10,16], and the knowledge of already well-established embedded/integrated psychological services at the hospital. The importance of taking a systemic approach was paramount, and, therefore, any psychological service provision was also offered to parents and siblings of the index child. The service was developed alongside the NICE guidelines for psychological trauma and includes both inpatient and outpatient psychological interventions.

**Figure 1:**
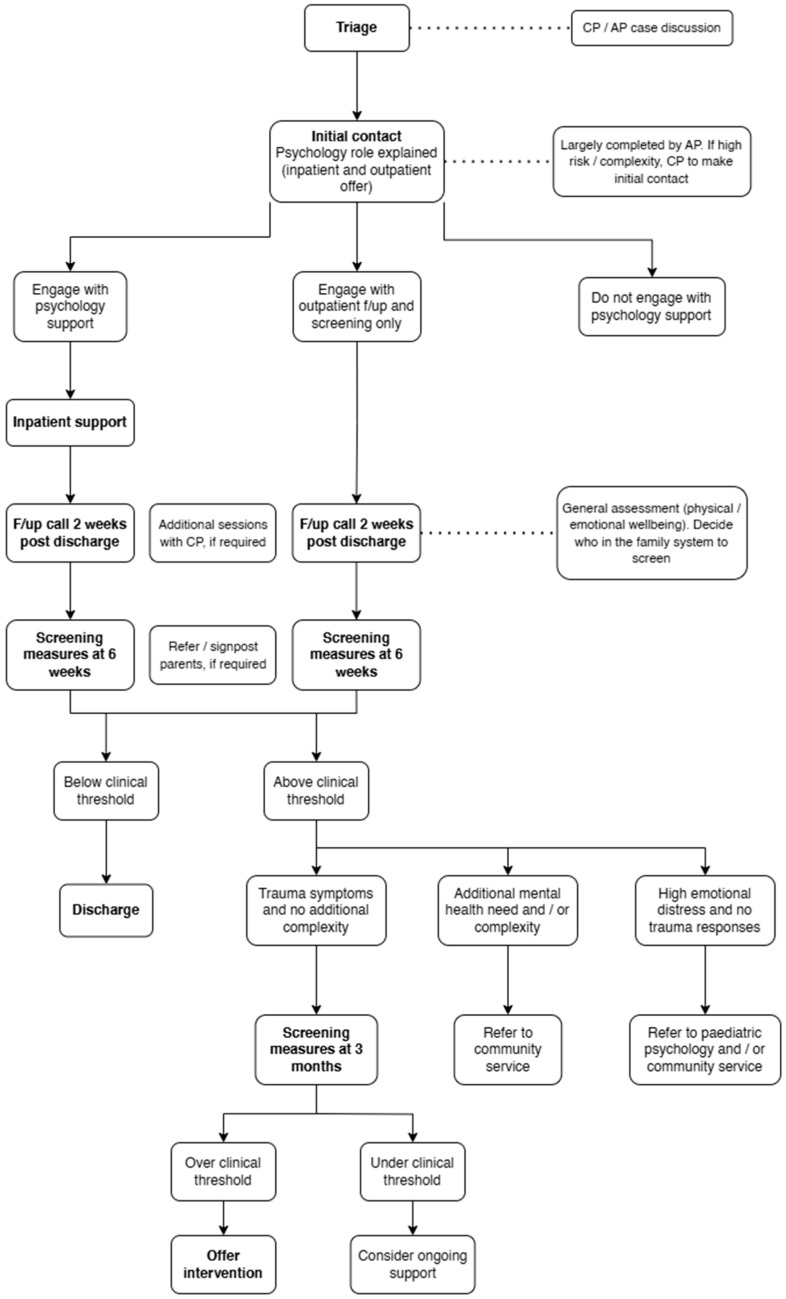
Flowchart detailing the paediatric major trauma psychology pathway (the dotted lines provide additional details at various time points).

Figure 1 outlines the key components of the offer to families of children admitted to wards.

### 2.5. Point of Admission

At the point of admission to a ward, families were informed about the psychological support available in the context of the trauma and the embedded nature of psychological professionals in the major trauma team.

### 2.6. Initial Triage (Completed by the Psychologist)

Initial triage involved a detailed review of available documentation on the clinical system about the child’s background, clinical history (physical and mental health), mechanism of injury, and circumstances surrounding their hospital admission, as well as conversations with the team involved in the child’s care (doctors, nurses, and youth workers).

During the first contact with families, the psychologist focused on engagement, destigmatising input, normalising emotional responses to hospital admission and, in particular, the psychological impact of trauma and, where appropriate, providing trauma psychoeducation.

For note, psychoeducation is a method for providing patients and families with theoretical and practical information to help understand and manage the consequences of their difficulties [17]. In this context, it often involved learning about common responses to trauma and how this might impact on CYPs’ emotions, behaviours, and wellbeing.

### 2.7. Inpatient Psychological Support

Specialist psychological assessment and intervention were offered proactively to all the children and families as a standard part of the inpatient and follow-up care. This involved working directly with children, young people, and families to manage physical and psychological symptoms, improve understanding of their care, facilitate complex decision making and MDT working. It also involved supporting the wider MDT to encourage psychological and psychosocial discussions or completing joint sessions with members of the MDT to enhance CYPs’ engagement in rehabilitation.

### 2.8. Outpatient Psychological Support

- Initial follow-up appointment (completed two weeks post discharge):

Two weeks following discharge, children and families were offered an appointment, virtually or in person, to discuss adjustment back home, considerations around school or other activities, as well as discussions in relation to physical and emotional health and wellbeing. This appointment was also used to determine who in the family system (e.g., parents/siblings) would benefit from being involved in the ongoing screening appointments.

From this point, families were offered up to 3 additional appointments with the clinical psychologist, often focusing on trauma stabilisation. Trauma stabilisation involves providing individuals with the skills to help stabilise and manage trauma symptoms [18].

ii. First screening appointment (six weeks post trauma):

Six weeks following the traumatic incident, a routine psychological screening appointment was completed with families. This was typically completed online or in person.

Details of the psychological screening measures used can be found in the measures section.

The outcome of the screening appointment determined the following:

Under 18s: Children and young people under the age of 18 years old, who were above the clinical threshold on the trauma measure, were kept open to the service for active rescreening. For children and young people not reporting trauma symptoms, but reporting high levels of distress, onward referrals to other appropriate services were considered.

Adults: For parents or siblings over 18 years old, who were scoring above the clinical threshold on the trauma measure and/or emotional wellbeing measure, onward referrals and signposting were discussed. These have typically involved referrals made to adult mental health, local talking therapies, or third-sector services. See Section 3, which outlines which services individuals were referred to.

iii. Repeat screening appointment (completed 3 months post trauma):

Children and young people were rescreened with the same measures 3 months following the trauma. For children and young people with persistent trauma symptoms, a specialist psychological/trauma-specific intervention was offered with the clinical psychologist.

### 2.9. Psychological Interventions

Direct psychological interventions offered by the major trauma psychologist were largely trauma-focused interventions, as outlined below. However, other direct psychological interventions included support around pain management, adjustment to injury and/or disfigurement, and management of ongoing anxiety or low mood. Indirect interventions included offering psychological formulation and education to the medical teams and liaison with local services to improve the psychological care of the child.

The following three trauma-focused interventions were routinely offered. Of note, these therapeutic interventions were not offered simultaneously but offered depending on the clinical need and fit with the clinical formulation.

- Trauma-Focused Cognitive Behaviour Therapy (TF-CBT): TF-CBT is an evidence-based intervention for individuals that have experienced a traumatic event and developed subsequent trauma-related symptoms [15,19,20].
- Eye Movement Desensitisation and Reprocessing (EMDR): EMDR is a form of psychotherapy developed in the late 1980s by Francine Shapiro [21]. Underpinned by the Adaptive Information Processing (AIP) model, the aim of EMDR is to support the processing of trauma memories.
- Storytelling/Narrative approach: Trauma disrupts memory processing [20,22]; storytelling approaches aim to put together coherent narratives for the child to connect to parts of their life [23].

### 2.10. Measures (Standardised Measures Are Accessible Online or by Request to the Author)

The psychological screening measures used were selected based on brevity and their validity for use with a paediatric population. The purpose of the measures was to identify any persisting psychological distress for children and families.

As this pathway is a pilot, different measures were administered during early stages to determine their appropriateness within this setting and population of children, young people, and families. The following screening measures were trialled:

#### 2.10.1. Children and Young People (0–17 Years Old)

Trauma measures:

- Preschool posttraumatic stress symptom inventory (PPSSI) (3–5 years old): The PPSSI is based on the DSM-IV criteria for PTSD and used for preschoolers. It is a 17-item questionnaire, administered by a clinician as a parent interview. Responses are recorded on a Likert scale from 0 to 2 (not present, present, present for more than 1 month). Its reliability for assessing for children’s fears is questionable (α = 0.69). However, it has been found to have acceptable–good reliability for all the other constructs, ranging between 0.75 and 0.86. When interpreting the scores, there is no established clinical cut-off point and so scoring is continuous [24].
- Children’s Revised Impact of Events Scale (CRIES-13) (3–17 years old): Children’s posttraumatic stress symptoms were measured using the 13-item version of the CRIES. This is a self-report measure for children aged 8 years and above. It has established reliability and validity (α = 0.80) [25]. A total score of 30 means that 75–83% of children with a PTSD diagnosis have been correctly identified [25].
- Child and Adolescent Trauma Screen (CATS) (3–17 years old): The CATS is based on the DSM-5 and screens for potentially traumatic events and symptoms in children and adolescents. It has been found to have good–excellent reliability, with a range between 0.88 and 0.94 [26]. The CATS is used for preschoolers, children, and adolescents. There is a self-report measure for 7–17-year-olds and two caregiver versions, one for 3–6 years old and one for 7–17 years old.

Of note, for children under the age of 3 years old, it was not possible to find an appropriate measure. Therefore, a semi-structured caregiver report was developed based on clinical experience, focusing on observable changes to the child’s mood, anxiety, behaviour, sleep, appetite, and physical symptoms. This can be found in Appendix A.

Emotional wellbeing measures

- Paediatric Index of Emotional Distress (PI-ED): Emotional distress, including anxiety and depression symptoms, was measured using the PI-ED screening measure [27]. The PI-ED is a 14-item self-report measure for children from 8 to 16 years old. It has established reliability and validity in the paediatric population (α = 0.86) [27]. A total score of 20 or above indicates a CYP experiencing clinically significant levels of distress [27].
- Hospital Anxiety and Depression Scale (HADS): This measure was used with children aged 17 years old, before the Patient Health Questionnaire-4 was introduced to the service. The HADS [28] is a self-assessment scale for detecting depression and anxiety in a hospital outpatient clinic. The HADS has been found to perform well in assessing the symptom severity of anxiety and depression [29].
- Patient Health Questionnaire-4 (PHQ-4): The PHQ-4 [30] is a brief screening measure to assess anxiety and depression in the general population and primary care. The scale produces a total score and two subscale scores: one for anxiety and one for depression (2 items per subscale). This measure has a Cronbach reliability of α = 0.87. This was used with 17-year-old children, who were too old to complete the PI-ED.

#### 2.10.2. Parents/Carers (+Siblings < 18 Years)

For parents and siblings over the age of 18 years, the following psychological screening measures were trialled:

Trauma measures:

- Impact of Events Scale Revised (IES-R): Posttraumatic stress symptoms were screened for in parents, using the IES-R. This is a self-report measure, used for adults over 18 years old, with established reliability and validity (α = 0.96). A cut-off score of 24 or more was used as an indication of ‘clinical concern’ for posttraumatic stress symptoms [31,32].
- The PTSD Checklist for DSM-5 (PCL-5): The PCL-5 [18] is a standardised self-report measure of PTSD symptoms. PCL-5 scores demonstrated strong reliability and validity with psychometric properties of internal consistency (α = 0.94), test–retest reliability (r = 0.82), and convergent (rs = from 0.74 to 0.85) and discriminant (rs = from 0.31 to 0.60) validities [33].

Emotional wellbeing measures:

- HADS (as described above).
- PHQ-4 (as described above).

### 2.11. Data

Descriptive statistics were used to describe the patient demographics, mechanism of injury, admission information, psychological screening scores, number of psychological support sessions attended following hospital admission, and onward referrals made.

## 3. Results

### 3.1. Demographics for All the Major Paediatric Major Trauma Cases

From 6 July 2023 to 15 July 2024, there were 205 paediatric major trauma calls. One-hundred thirty-seven were admitted to a ward for an inpatient stay, fifty-six were not admitted to a ward, six were transferred to another hospital, and six passed away. The current analysis focuses on the 137 patients admitted to a ward.

Demographic data on all the major trauma cases are presented initially. Unfortunately, because of missing ethnicity data in the electronic patient records, it was not possible to report on ethnicity data for all the major trauma cases. Instead, ethnicity data are reported for cases on the pathway, as these were routinely collected at this point.

#### 3.1.1. Age (All Paediatric Major Trauma Cases)

Of the 137 children and young people admitted to a ward, roughly one-third (32%) were between the ages of 15 and 17 years and one-quarter (26%) were between 12 and 14 years.

Additionally, 14% were between 9 and 11 years, 8% were between 6 and 8 years, 5% were between 3 and 5 years, and 15% were between 0 and 2 years.

#### 3.1.2. Gender (All Paediatric Major Trauma Cases)

The majority of the paediatric major trauma calls were male (72% male and 28% female).

#### 3.1.3. Mechanism of Injury (All Paediatric Major Trauma Cases)

Mechanism of injury categories have been taken from the Trauma Audit and Research Network (TARN) database. TARN, established in 1990, is the national clinical audit for trauma care across NHS England.

**Figure 2:**
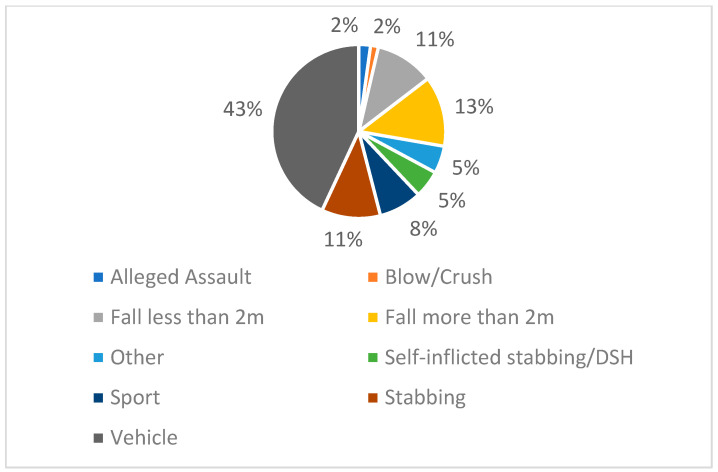
Pie chart showing the mechanisms of injury.

Figure 2 shows the mechanisms of injury. The highest number of injuries seen during this period was those involving a vehicle (43%). Following this, falls more than 2 metres (13%) and less than 2 metres (11%), stabbings (11%), sports (8%), other (5%), self-inflicted stabbings/deliberate self-harm (DSH) (5%), alleged assaults (2%), and blows/crushes (2%).

### 3.2. Psychological Data

Since the initiation of the pathway, 129 out of the 137 patients admitted to a ward (94%) were offered psychological support. This is a notable increase from the time prior to psychological service provision being embedded in the MTC, when only 20% of the cases were seen by a psychologist. The eight patients who were not offered psychological support proactively were admitted to a ward during the initial pathway development phase and, as such, were only able to access psychological support on an MDT referral basis.

**Figure 3:**
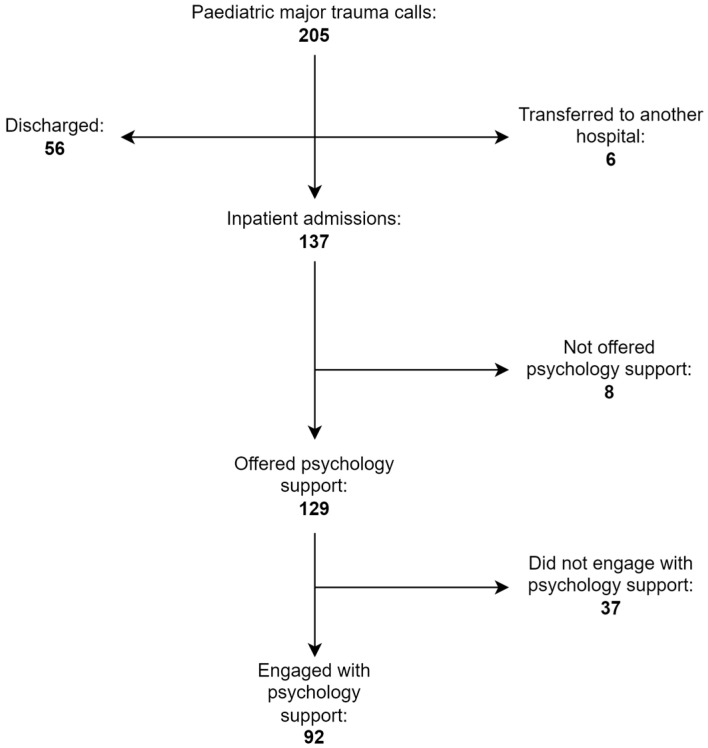
Flowchart showing the numbers of CYP and families admitted to hospital and those that engaged with psychological support.

Seventy-one percent (92/129) of the patients and families with psychological support during the inpatient admission engaged with ongoing psychological support follow-ups (see Figure 3).

#### 3.2.1. Ethnicity (Seen by Psychology)

Of the children and young people seen by the psychology team, the largest group identified as white (36%). Fifteen percent identified as black or black British, thirteen percent identified as ‘other’, ten percent identified as Asian or Asian British, ten percent identified as mixed, and sixteen percent did not know/not state an identity.

#### 3.2.2. Gender (Seen by Psychology)

Seventy-one percent of the CYP seen by the psychological support service were male and twenty-nine percent were female.

#### 3.2.3. Psychological Screening Appointments (6 Weeks Post Incident)

A total of 103 screening appointments were completed 6 weeks after the traumatic incident. Fifty-two were CYP, five were siblings, and forty-six were parents/carers (N = 103).

The results below will just report on those children, young people, and parents/carers with standardised scores (N = 92).

#### 3.2.4. Missing Data for 6-Week Screening

Before the CATS was introduced as a routine screening measure for younger children, 10 children under 3 years old had parent/carer-reported measures conducted. As noted above, these consisted of either self-developed semi-structured interview questions or the PPSSI. Neither measure yielded a total score and, therefore, these CYP were not included in the graph below.

**Figure 4:**
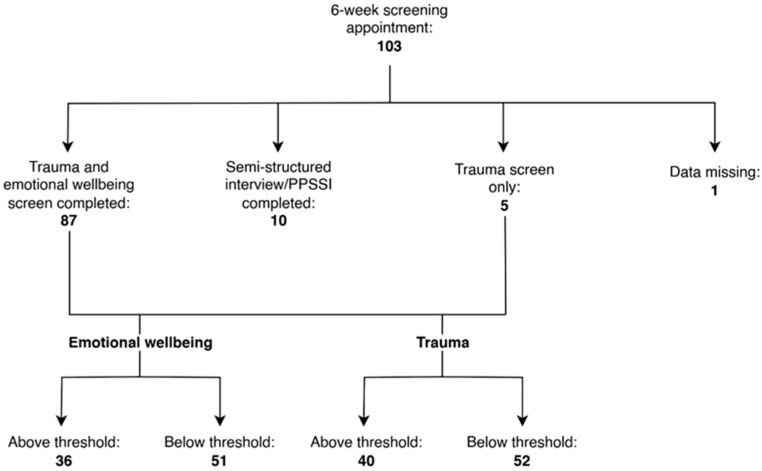
Flowchart showing the numbers of CYP, parents/carers, and siblings screened and the results.

One child had missing screening data. Four children and one parent completed the trauma screening questionnaire but not the emotional wellbeing measure, and eighty-seven (children, parents/carers, and siblings) completed both the trauma and emotional wellbeing screenings (see Figure 4).

Descriptive statistics and normality tests for the questionnaires used 6 weeks following the trauma, for all the participants (index CYP, parents/carers, and siblings), are shown in Table 1. Participants reported moderate levels of posttraumatic stress in the IES-R (M = 30.55, SD = 23.02), with a high degree of variability. Mild levels of anxiety and depression were shown in the PHQ-4 (M = 3.97, SD = 3.70), with a wide spread of scores. Participants displayed high scores for anxiety and depression in the HADS (M = 15.41, SD = 12.73), with considerable variability. The data showed approximately normal distributions, with skewness and kurtosis values within acceptable ranges (±2).

Table 2 displays the correlation matrix for the study questionnaires. Spearman’s correlation was chosen, as the variables were measured on an ordinal scale. Some correlations are missing because participants completed two specific questionnaires based on their age, resulting in incomplete data for certain questionnaire pairs. Several significant correlations were observed: PI-ED scores were positively correlated with CATS scores (r = 0.71, *p* < 0.01) and CRIES-13 scores (r = 0.48, *p* < 0.05). Additionally, HADS scores were positively correlated with CRIES-13 scores (r = 1.00, *p* < 0.01) and IES-R scores (r = 0.74, *p* < 0.01). Finally, PHQ-4 scores were negatively correlated with CRIES-13 scores (r = 1.00, *p* < 0.01) and positively correlated with PCL-5 scores (r = 0.83, *p* < 0.01). All the other correlations were non-significant.

#### 3.2.5. Outcomes of Psychological Screening at 6 Weeks

**Figure 5:**
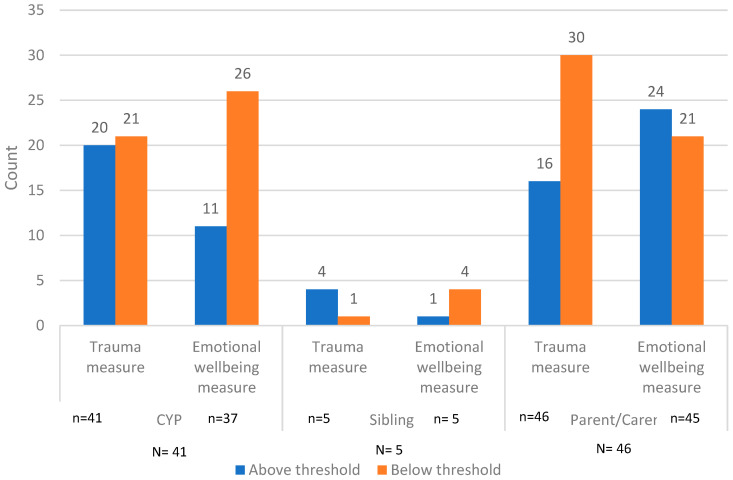
Outcomes of 6-week screening (children, siblings, and parents/carers) (N = 92).

Figure 5 details the numbers of CYP, siblings, and parents/carers who fell above or below clinical thresholds on the screening measures completed 6 weeks following the trauma. Twenty CYP, four siblings, and sixteen parents scored above the cut-off point on the measure of trauma, whilst eleven CYP, one sibling, and twenty-four parents scored above the cut-off point on the measure of mood and anxiety, which is indicative of emotional distress.

#### 3.2.6. Parent–Child Data Pairs

Outcome data were available for 16 mother and CYP pairs. Sixty-three percent of the mothers scored in the same category as their child for trauma symptoms (i.e., above or below the clinical threshold). There were 10 father and CYP pairs, and 50% of the fathers scored in the same range as their child on the trauma measure.

The lower number of parent–child pairs of data is reflective of cases where either only the child or only a parent engaged with psychological screening and support. They also include children who may have been too young for a standardised measure at that time (i.e., before the CATS was introduced).

#### 3.2.7. Additional Psychological Support Appointments

As a part of the pathway, all the CYP and their families were offered psychological input during the inpatient admission and following discharge, if required (outside of the routine screening appointments). Largely, these sessions were provided by the clinical psychologist and focused not only on trauma psychoeducation and trauma stabilisation but also (as needed) on adjustment to disability and/or visible difference and mood management.

Nineteen CYP accessed 1–3 follow-up appointments, four accessed 4–6, and one accessed more than 14 follow-up appointments.

Twenty-nine parents/carers and four siblings accessed 1–3 follow-up appointments, four parents/carers accessed 4–6, and one parent/carer accessed more than 14 follow-up appointments. Of note, the CYP and parents who required more than 14 sessions tended to have longer inpatient stays. Table 3 shows the numbers of clinical contacts received outside of the routine screening appointments.

#### 3.2.8. Referrals to External Services

Of the 41 CYP, 16 were kept open to the team to actively rescreen at 3 months. Four were referred to CAMHS, and three were referred to another external service for psychological support; one was referred for school-based support, one had existing support in place elsewhere by the time of the screening, and sixteen were discharged.

Of the 46 parents/carers, 18 were referred to external services, mostly local talking therapy services. Two parents were receiving support elsewhere at the time of the screening, and twenty-six were discharged.

Of the five siblings, four were kept open to the team to rescreen at 3 months, and one was discharged. No siblings were referred for support externally (see Table 4).

#### 3.2.9. Outcomes of the 3-Month Screening Appointment

**Figure 6:**
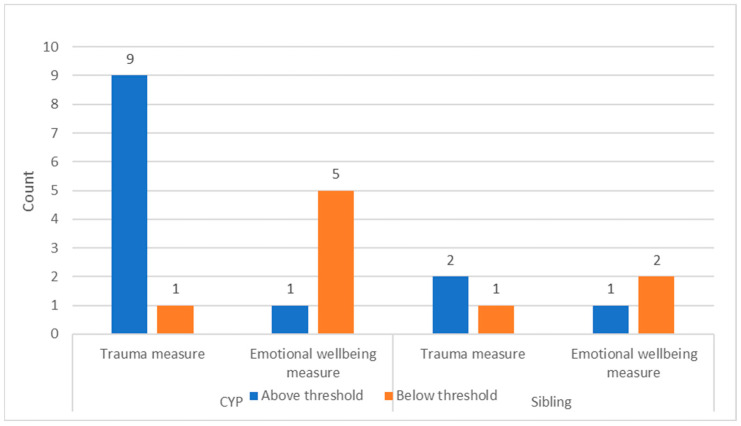
Outcomes of 3-month screening (children and siblings) (N = 13).

Children who scored above the clinical threshold on the trauma measures at the 6-week screening appointment were actively offered further screening at 3 months post incident. This was used to identify those people who may require a trauma-specific intervention. Ten children and three siblings went on to complete a 3-month screening appointment (see Figure 6).

The lower numbers for the 3-month screening reflect children who were either referred elsewhere in the interim period (e.g., to CAMHS), disengaged from the service (between the 6-week and 3-month screenings) or went on to complete a 3-month screening later because of their physical health requirements (and, therefore, not in the timeframe reported in this paper).

## 4. Patient Feedback

Patient feedback was sought regularly to help shape the development and design of the pathway. As standard, this was collected through the use of experience-of-service questionnaires at the point of discharge, as well as through ad hoc qualitative feedback from children, young people, and their families.

Feedback forms could be accessed online and were based on standardised service-user forms, such as the Chi-ESQ [34], Family Needs Questionnaire—paediatric version [35], and Therapy Satisfaction Questionnaire for 5–11-year-olds [36]. Four age-appropriate feedback forms were created in order to ensure accessibility to CYP across the age range (5–8 years old, 9–11 years old, 12–17 years old, and parent/carer feedback). All the forms consisted of both closed (Likert scale) and open questions to elicit information about different aspects of the care they received.

Overall, the feedback received from the children, young people, and families indicated that the service met their needs well, and the feedback was overwhelmingly positive about their experience of care from psychological support. Please see Appendix B for closed responses collected from the CYP, siblings, and parents/carers to date. Brief extracts from open-ended questions have been detailed below (see Table 5).

## 5. Discussion

This paper presents the audit data collected following the design and implementation of a new integrated psychological care model at a tertiary paediatric major trauma centre. The first year of the project saw a significant increase in the numbers of children and families offered and accessing psychological support following an admission to a paediatric ward under the major trauma team (an increase from 20% of the cases between 2022 and 2023 to 94% of the cases between 2023 and 2024).

Of the 94% of the families offered psychological support, 71% actively engaged with the psychology pathway, meaning that far higher numbers of children, young people, and families were receiving psychological support during the acute stage post incident, as well as following discharge (including accessing proactive screening for the early identification of ongoing emotional distress). The proactive approach to offer psychological support in this way, embedded as a part of the medical team, aligns with a growing evidence base for systemic healthcare screening, which has been seen to produce significant effects at a population level in various healthcare sectors [37,38].

### 5.1. Patient Demographics

The majority of the major trauma calls were males (72% male and 28% female) between the ages of 15 and 17 years old (32%), which coincides with previous research on cases of adolescent trauma admissions [39].

The highest number of injuries seen involved a vehicle (43%), which mirrors prior research identifying RTCs as leading aetiologies for young people between 10 and 24 years old [39,40]. Our sample saw similar rates of trauma related to violence, with stabbings at 11%; this is compared to 10.2% reported in a published article from the TARN database between 2008 and 2017 [39]. It is known that homicide is the third highest killer in males, in England and Wales, aged 18 years and under, following suicide and traffic collisions [40].

Ethnicity data on young people’s medical records were sparse and often missing. Although the psychology team proactively asked questions about a family’s ethnicity, this meant that for those families who opted out of psychological support involvement, ethnicity data were not available. This coincides with prior research in England and contributes to a misleading picture of health inequalities [41]. Of the families seen by the psychology team, the largest proportion of families identified as being white (36%), black or black British (15%), other (10%), Asian or Asian British (10%), or mixed (10%), and 16% were not stated.

### 5.2. Trauma Stabilisation Sessions

When looking at the direct psychological interventions offered, the highest number of additional sessions required (outside of the follow-up and screening appointments) was between one and three sessions. Lower numbers of young people and families received longer-term support from the clinical psychologist prior to a trauma-specific intervention. The needs of these young people were reflective of those who sustained significant medical injuries that required longer-term hospital admissions and were faced with greater adjustment to their injuries, emotional distress, and complex medical decision making.

### 5.3. Presence of Trauma Symptoms in Children

The results from this audit demonstrate that half the children and young people screened at 6 weeks scored above the clinical threshold on the trauma measure (49%), whilst 30% scored above the clinical threshold on the Pi-Ed, indicative of emotional distress. Of these children, 22% of the children reported persistent trauma symptoms at the three-month screening, and 3% continued to score above the clinical threshold for emotional disturbance. It is important to note that this number does not include children and young people who were referred in the interim to community services (CAMHS and third-sector organisations) because of additional or pre-existing mental health difficulties or young people with a high level of contextual safeguarding concerns. Therefore, the authors would hypothesise that the number of those with persistent psychological symptoms at 3 months post incident may well be higher than it is possible to report herein. The literature regarding PTSD following major trauma ranges from 27 to 36% [42,43,44]. However, studies have tended to be based on adult trauma patients, and there is less research on PTSD prevalence following major trauma in a paediatric population. The numbers presented in this audit are largely in line with the above adult research and form a basis from which further research in paediatrics could be conducted.

In the current service model, children with persistent trauma responses went on to complete a trauma-focused intervention with the clinical psychologist (EMDR, TF-CBT, or narrative). Given the significant pressures faced by CAMHS across the UK and waiting times known to be at least 2 years long [45], this integrated approach to care allowed for timely and proactive psychological support (including therapy) to be offered to children 3 months following the trauma. This early intervention approach aims to mitigate, and ultimately prevent, worsening outcomes for this population of children and young people and aligns with research on prevention and early intervention for youth mental health [46].

The current number of patients and variety of measures used in screening appointments during the early stages of the pathway’s development mean that statistical analysis looking at predictive and risk factors for psychological outcomes is not possible at the present time. This is one of the main limitations of the current evaluation. The authors hypothesise that the provision of additional psychological support to children and families (in particular, trauma psychoeducation and trauma stabilisation) may have had the positive effect of reducing the extent and/or development of PTSD over the longer term.

Some established research has indicated that PTSD after major trauma is not related to the measure of the injury severity but to other factors, including the child’s appraisals of the trauma [7], for example, blame cognitions [42], experiences of prior trauma, and the nature of the posttraumatic social support [47]. As already stated, it is not yet possible to draw conclusions based on predictive factors, given the small sample to date, but it is the team’s intention to investigate this, along with other predictors of outcomes, with a fuller dataset.

### 5.4. Parental and Sibling Trauma Symptoms—The Importance of a Systemic Approach

A high number of parents and carers (39%) who engaged with the screening appointments were signposted to external agencies for therapeutic support, namely, local talking therapies. This demonstrates the high level of systemic need following paediatric major trauma. It is well known that parents can be affected by children suffering trauma and hospital admissions [48,49], and parental distress can impact psychological and physical trajectories for children [50].

By taking a systemic approach to the early identification of distress through screening, trauma stabilisation, and actively making referrals to community services for parents, this allowed for a larger proportion of the family to be supported psychologically, with anticipated long-term benefits to the index child.

An especially novel aspect of this integrated model is that siblings of the index child were also included in the screening and treatment pathway if clinically indicated, e.g., if the support system around the sibling expressed concerns about their wellbeing or they witnessed the traumatic incident. Five siblings were screened at 6 weeks, and three siblings were included in the 3-month screening appointment, going on to receive psychological intervention within the team. This is a unique service offer and one that is less likely to be available as standard through mental health services, such as CAMHS, meaning that there is the added potential of a service like this mitigating the wider impact of a traumatic event and maximising the long-term health, social, and educational benefits for the whole family.

## 6. Clinical Implications

As it stands, the authors believe this integrated model provides a robust approach to the early identification of distress (via screening) and the active treatment of psychological needs in children and young people, following an admission to a tertiary hospital for a major trauma. In particular, taking a proactive approach has enabled CYP who would otherwise be less likely to access psychological care to receive input. This is evidenced in the much larger proportion of children affected by community violence and those who are admitted following acts of deliberate self-harm engaging in the pathway and receiving psychological care. Embedding psychological support into the MTC has also meant that there have been enhanced opportunities for sharing psychological formulation, teaching, training, and indirect psychological interventions—all of which are likely to have improved holistic care and outcomes for CYP in the service.

Clinical and anecdotal observations have identified three potential subgroups within the population of CYP under the care of the psychology team. One group of CYP are those experiencing single-incident traumas (e.g., traffic accidents and sporting injuries). These young people were distinct in their demographics (mechanisms of injury) and psychological needs (anxiety, pain management, and ongoing trauma symptoms) compared to CYP who were either admitted as a result of community violence or for those who were admitted following deliberate acts of self-harm (falls from more than 2 m and other suicide attempts). It was observed that these latter two groups more frequently had a number of other complex safeguarding, mental health and/or social needs that were important facets of their care. This poses unique challenges to clinicians not only in terms of maintaining engagement but also meeting the complex needs of vulnerable young people. Individual work still included trauma psychoeducation and stabilisation, as well as psychological support for low mood, anxiety, and pain management tailored to young peoples’ individual needs and circumstances. However, as noted above, psychological support and interventions required creativity in the approach. It often needed to be delivered through joined multiagency work, including connecting with the system around the child, such as CAMHS, home treatment teams, children’s social care, and community organisations, such as RedThread and Off the Record. Working with these CYP also highlighted the importance of destigmatising psychology and, therefore, the benefits of being seen as a core member of the treating team to help support engagement.

## 7. Limitations and Future Research

There are a number of limitations and areas for future research. As already stated, it was not possible to proactively screen CYP who were not admitted to a ward and who were discharged straight from the Emergency Department. Ideally, and with more resources, it would be good to include this group of CYP in the proactive screening offer. One possible screening tool, developed in the US [51], could be an interesting area for further exploration in relation to its applicability in a UK setting.

In addition, at the time of writing, because of the variability in the standardised measures used in the pilot phase, there were insufficient numbers of CYP to carry out any statistical analysis on the screening data collected. This is an area for further research and would help to identify the predictive risk and protective factors affecting psychological outcomes for CYP, following admission for major trauma, and whether this aligns with research already published in this area [7,10].

It is also hoped that there is a way of better capturing the varied ways of working with CYP who present with different mechanisms of injury, in particular, by evaluating the indirect psychological intervention offered to those with complex safeguarding, social care, and mental health needs, and the added benefits of active trauma psychoeducation and stabilisation work.

Moving into the second year of offering the integrated model, the authors wish to continue to strengthen connections with community teams and third-sector organisations who can better support CYP post discharge. This is particularly important for building support to CYP who require outreach or psychological care closer to home and to continue to review how to offer evidence-based psychological interventions that meet the needs of the CYP affected by community violence, where there could be a considerably high level of ongoing contextual risk [26,44].

The long-term follow-up, as well as a better understanding of the predictive and risk factors of all the patients, is an essential part of any ongoing research in this area.

## 8. In Summary

A proactive and integrated service model allows for more children, young people, and families to be offered psychological support following an admission for major trauma. This includes a greater number of CYP accessing early identification of distress, through screening, and psychological intervention as needed.

By offering outpatient support as a part of the model of care, families can be monitored following trauma and safety-netted where needed.

Trauma stabilisation and psychoeducation sessions could be having a preventative impact on the development of PTSD for children and families.

Trauma-reprocessing therapy can be offered to children and siblings in a shorter timeframe than in community services, given the current pressures faced by the NHS. Early intervention is likely to have positive impacts on the trajectories of children and young people’s recoveries from major traumas, including from physical, psychological, and social perspectives. This also applies to their siblings and parents.

A systemic approach to service delivery is essential if we are to best meet the needs of CYP following a major trauma.

Further research is needed to explore the differing predictive factors that might account for psychological outcomes in this population, accounting for the differing mechanisms of injury and associated psychological and social factors.

## Figures and Tables

**Table 1:** Descriptive statistics for study outcome measures.

Questionnaire Type	Questionnaire	N	Mean	SD	Minimum	Maximum	Skewness	Kurtosis
Trauma	CATS	22	16.86	13.36	1.00	44.00	0.77	−0.20
	CRIES-13	24	18.33	11.22	0.00	40.00	0.55	−0.32
	PCL-5	26	21.04	17.54	0.00	67.00	0.91	0.22
	IES-R	20	30.55	23.02	4.00	76.00	0.61	−0.92
Emotional wellbeing	PI-ED	34	12.91	7.50	0.00	34.00	0.63	0.49
	PHQ-4	31	3.97	3.70	0.00	11.00	0.43	−1.26
	HADS	22	15.41	12.73	0.00	42.00	0.54	−0.56

**Table 2:** Spearman correlations between study questionnaires.

Questionnaire	1	2	3	4	5	6	7	N
1. CATS	—	-	-	-	0.71 **	−0.50	-	19
2. CRIES-13	-	—	-	-	0.48 *	−1.00	1.00 **	23
3. PCL-5	-	-	—	-	-	0.83 **	-	26
4. IES-R	-	-	-	—	-	-	0.74 **	19
5. PI-ED	0.71 **	0.48 *	-	-	—	-	-	34
6. PHQ-4	−0.50	−1.00 **	0.83 **	-	-	—	-	31
7. HADS	-	1.00 **	-	0.74 **	-	-	—	22

Note: *p* < 0.05 (*); *p* < 0.01 (**). Missing correlations (denoted by ‘-’) were because of incomplete data from participants. The correlation of a questionnaire with itself is represented by ‘—’, as this will always be 1.00.

**Table 3:** Numbers of clinical contacts received outside of the routine screening appointments.

	0	1–3	4–6	7–9	14+
CYP	13	19	4	3	1
Parents/Carers	11	29	4	1	1
Siblings	1	4	0	0	0

**Table 4:** Outcomes of the 6-week review for the index CYP, parents/carers, and siblings (N = 92).

	3-Month Screening	CAMHS Referral	School-Based Support	Referred Elsewhere	Existing Support Elsewhere	Discharged
CYP	16	4	1	3	1	16
Parents/Carers	0	0	0	18	2	26
Siblings	4	0	0	0	0	1

**Table 5:** Extracts of open-ended feedback collected from all the age groups and parents/caregivers.

Group	Quote
Children/Young people (5 years–17 years)	“They made me happy.”
	“I don’t think about the incident as much.”
	“They were able to help me through the difficulties of school.” “Seeing a psychologist made a big positive difference to my life, as it helped me process and cope with everything, making everyday life and having hospital visits so much easier. It has made my life much more positive again since my trauma.”
	“She helped me calm my worries in other areas of life and see how connected they all were.”
Parents/Carers	“It helped me get through the most difficult time of my life.”
	“It was reassuring to have the incident looked at across the family, not only how the event impacted us but also our capacity to support her.”
	“I found it really helpful that I was able to see the same person who I’d seen whilst my daughter was in hospital; it really helped me relax and trust.”
	“Phenomenal service; it was an unexpected service after the accident, and we felt very supported; expectations exceeded.”

**Figure A1:**
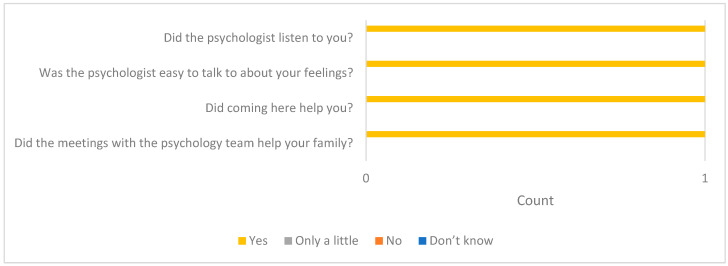
Responses to closed questions on the service feedback form for 5–8-year-olds.

**Figure A2:**
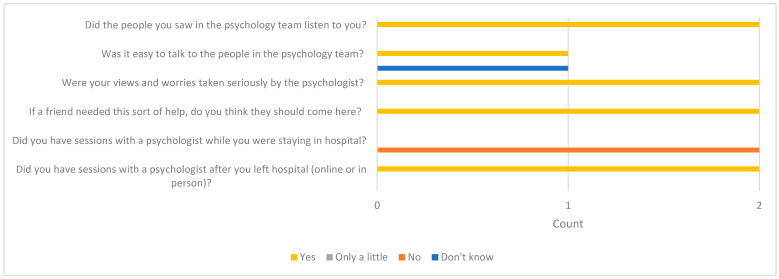
Responses to closed questions on the service feedback form for 9–11-year-olds.

**Figure A3:**
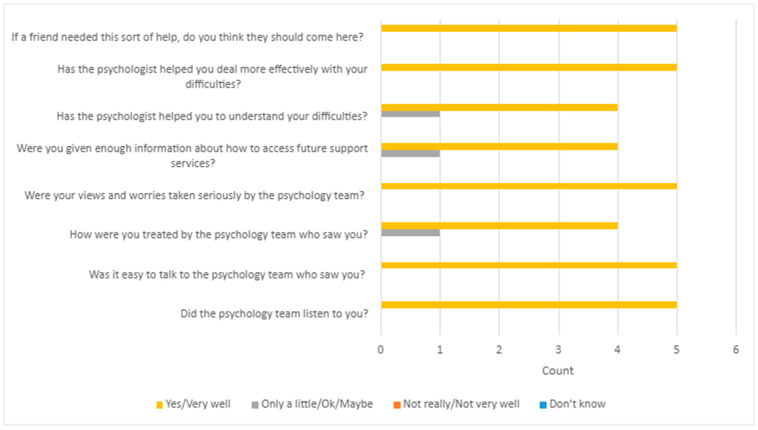
Responses to closed questions on the service feedback form for 12–17-year-olds.

**Figure A4:**
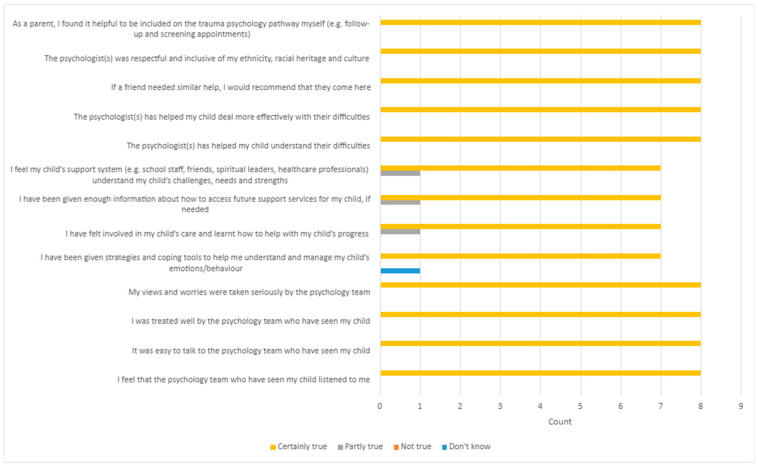
Responses to closed questions on the service feedback form for parents and caregivers.

## Data Availability

The data presented in this study are available on request from the corresponding author. The data are not publicly available because of privacy reasons.

## Appendix A. Caregiver Questions for Children Under 3 Years Old

1. Have you noted any concerns/observed changes in the following areas:
  - Mood/anxiety
  - Irritability
  - Tearfulness
  - Difficulties in separating
2. Behavioural changes. Have you noticed any concerns/observed changes in the following:
  - Withdrawal
  - Change in feeding
  - Change in behaviour towards parent/carer
  - Loss of interest in things they previously enjoyed
  - Other changes in behaviour
3. Sleep (concerns/observed changes)
  - Changes in sleeping pattern
4. Physical symptoms (concerns/changes)
  - Any physical symptoms

## Appendix B. Closed-Question Patient Feedback

### Appendix B.1

Five-to-eight-year-olds’ feedback:

This form contains six questions. Four of these are closed questions (Likert scale with visual aids—response options were “Yes”, “Only a little”, “No”, and “Don’t know”), and two are open ended, asking about positive and negative aspects of the care they received. To date, one child in this age bracket has completed the feedback questionnaire. The closed questions related to whether they felt listened to, whether it was easy to talk about their feelings, and whether the service helped them and their family. The child responded “Yes” to all these questions.

### Appendix B.2

Nine-to-eleven-year-olds’ feedback:

This form contains 13 questions plus demographic questions. Six questions are closed, and seven are open ended. Four of the closed questions are about whether they felt listened to, whether it was easy to talk about their feelings, whether their views and worries were taken seriously, and whether they would recommend the service to a friend. Responses were given on a four-point Likert scale consisting of “Yes”, “Only a little/Maybe”, “Not really”, and “Don’t know”. To date, two young people in this age range have completed the questionnaire. Aside from one child responding, “Don’t know” relating to whether it was easy to talk to the psychologist about their feelings, all the other responses were “Yes”.

### Appendix B.3

Twelve-to-seventeen-year-olds’ feedback:

This form contains 18 questions and demographic questions. Nine questions are closed questions, and nine are open ended. To date, five young people in this age range have completed the questionnaire. See Figure 5 for the responses to the closed questions, excluding two questions about whether they were seen as an inpatient and/or an outpatient.

### Appendix B.4

Parent/caregiver feedback:

This form contains 19 questions and demographic questions. Thirteen are closed questions, and six are open ended. To date, eight parents and caregivers have completed the questionnaire. Figure A4 shows the responses to the closed questions.
